# Protein sequence alignment with family-specific amino acid similarity matrices

**DOI:** 10.1186/1756-0500-4-296

**Published:** 2011-08-16

**Authors:** Igor B Kuznetsov

**Affiliations:** 1 Cancer Research Center, Department of Epidemiology and Biostatistics, University at Albany, State University of New York, One Discovery Drive, Rensselaer, NY, USA 12144

## Abstract

**Background:**

Alignment of amino acid sequences by means of dynamic programming is a cornerstone sequence comparison method. The quality of alignments produced by dynamic programming critically depends on the choice of the alignment scoring function. Therefore, for a specific alignment problem one needs a way of selecting the best performing scoring function. This work is focused on the issue of finding optimized protein family- and fold-specific scoring functions for global similarity matrix-based sequence alignment.

**Findings:**

I utilize a comprehensive set of reference alignments obtained from structural superposition of homologous and analogous proteins to design a quantitative statistical framework for evaluating the performance of alignment scoring functions in global pairwise sequence alignment. This framework is applied to study how existing general-purpose amino acid similarity matrices perform on individual protein families and structural folds, and to compare them to family-specific and fold-specific matrices derived in this work. I describe an adaptive alignment procedure that automatically selects an appropriate similarity matrix and optimized gap penalties based on the properties of the sequences being aligned.

**Conclusions:**

The results of this work indicate that using family-specific similarity matrices significantly improves the quality of the alignment of homologous sequences over the traditional sequence alignment based on a single general-purpose similarity matrix. However, using fold-specific similarity matrices can only marginally improve sequence alignment of proteins that share the same structural fold but do not share a common evolutionary origin. The family-specific matrices derived in this work and the optimized gap penalties are available at http://taurus.crc.albany.edu/fsm.

## Background

Pairwise alignment of amino acid sequences is a cornerstone sequence comparison method used in a variety of computational applications [1-4]. A mathematically rigorous and computationally efficient way of finding optimal global [5] and local [6] alignments for a given pair of sequences is provided by dynamic programming. The outcome of a dynamic programming procedure applied to align amino acid sequences critically depends on the alignment scoring function used by this procedure [7,8]. Therefore, for a specific alignment problem one needs a way of selecting the best performing scoring function [7-9]. The traditional alignment scoring function most commonly used in dynamic programming consists of an amino acid substitution matrix and gap penalties [5,6]. Recently, several novel sequence alignment algorithms have been developed that use scoring functions based on Hidden Markov Models (HMMs) [9-15]. On one hand, the probabilistic nature of these algorithms makes them superior to the substitution matrix-based alignment. On the other hand, they require estimation of a large number of transition and emission probabilities, which makes obtaining reliable HMMs especially tricky in the case of global alignments and small sequence datasets [16,17]. Despite the advent of more sophisticated alignment algorithms, global pairwise alignment based on amino acid substitution matrices still remains the well-established workhorse of sequence analysis. It is widely used for basic sequence comparison tasks that include the identification of structurally equivalent positions in homology modeling, which crucially depends on the quality of alignment between the target and template sequences [3], and in popular multiple sequence alignment algorithms, such as CLUSTAL [18] and TCOFFEE [19]. Therefore, improving the quality of substitution matrix-based global pairwise alignments is an important step in improving other more complex computational applications.

In substitution matrix-based alignment, matrix selection is the most important decision the user has to make because once a matrix is selected the values of its elements cannot be easily changed, unlike gap penalties. Most amino acid substitution matrices are similarity matrices based on the same underlying idea: they attempt to account for the similarity between two amino acids by computing how often these amino acids occur in the equivalent sequence positions in related proteins. It is assumed that if two amino acids *i *and *j *are often observed in the equivalent positions, they have similar biochemical properties and can be substituted one for another in the course of protein evolution. The differences between various similarity matrices are mainly determined by what groups of protein sequences are used to derive them and how the equivalent positions are defined. The mainstream matrices routinely utilized in sequence comparison, such as BLOSUM [20] and PAM [21], are obtained by counting the frequencies of amino acid substitutions observed in the columns of multiple sequence alignments of evolutionary related proteins. As an alternative to using multiple sequence alignments, one can count the frequencies of amino acid substitutions observed in the structurally equivalent positions of structurally similar (but not necessarily sequence-similar) proteins [22-24]. In this case, the structurally equivalent positions are identified by means of a computational technique known as structural superposition. Structural superposition detects the structurally-equivalent regions in two protein structures by using their geometrical properties only [25-28]. The amino acid sequence alignment that corresponds to the optimal structural superposition is generated after the superposition is complete.

The advantage of structural superposition is that it allows one to obtain high quality reference sequence alignments for both distantly related homologous proteins and for proteins that share the same structural fold but no common evolutionary origin. Such structure-based reference alignments can be used not only to derive amino acid similarity matrices but also to design benchmarks for assessing the performance of sequence alignment algorithms [29-33]. In a sequence alignment benchmark, the quality of sequence alignments produced by a given alignment algorithm is assessed by comparing them to the reference alignments and calculating the percentage of correctly aligned positions. Typically, the average percentage of correctly aligned positions is used to compare two or more alignment algorithms and the algorithm with the highest average is identified as the top-performing one. One problem with such an approach based on a simple comparison of the averages is that it lacks statistical testing and a larger average can come at the expense of a larger variability, especially in smaller datasets, thus not really reflecting a statistically significant difference. Another common problem with benchmarking sequence alignment algorithms is that it is usually performed on a large pooled sequence dataset that contains many diverse protein families. The use of pooled sequences leads to the loss of family-specific information and may bias the results towards the overrepresented protein families [7].

Virtually all existing amino acid similarity matrices are general-purpose matrices, meaning that they were derived by averaging substitution frequencies over many diverse protein families that represent the entire protein universe. General-purpose similarity matrices are required for such task as a sequence database search because in this procedure a query sequence is aligned with millions of diverse database sequences. However, in tasks such as a global sequence alignment only related sequences are typically used and it is often known in advance which protein family these sequences belong to. In such a case, even the best general-purpose matrix may not perform equally well on all protein families, because family-specific substitution patterns were mostly averaged out. For instance, it has been shown that amino acid similarity matrix derived for a specific protein family tends to perform better on proteins from this family than BLOSUM matrices [34,35]. The recent advances in the experimental data acquisition have provided a wealth of sequence and structural data that allow us to obtain reference sequence alignments for many diverse protein families and structural folds [33]. These reference alignments can be used to derive protein family- and fold-specific similarity matrices and to conduct family- and fold-specific performance evaluations.

In this work, I focus on the issue of finding optimized amino acid similarity matrices for pairwise global sequence alignment. I use a comprehensive set of reference sequence alignments to design a quantitative statistical framework for evaluating the performance of alignment scoring functions on protein family and structural fold levels and apply this framework to study the utility of family- and fold-specific amino acid similarity matrices for global sequence alignment. The results of this work indicate that the quality of pairwise global sequence alignment can be significantly improved by using family-specific similarity matrices.

### Dataset

I used the release 1.65 of the Sequence Alignment Benchmark (SABmark) [33] to derive protein family-specific and fold-specific amino acid similarity matrices and to evaluate the accuracy of pairwise sequence alignments obtained using different scoring functions. SABmark is a large general-purpose "gold standard" database specifically designed to evaluate the performance of pairwise sequence alignment algorithms. It consists of groups of the reference pairwise sequence alignments obtained from the consensus structural superposition of high-quality protein structures that cover the entire SCOP database [36]. All sequences in SABmark are divided into two sub-sets, "the Superfamily" sub-set (SUP) and "the Twilight Zone" [37] sub-set (TWI). Each SUP group contains homologous single-domain protein sequences with low to moderate degrees of sequence identity that belong to the same SCOP super-family. Each TWI group contains single-domain protein sequences that belong to the same SCOP fold and share no detectable sequence similarities (meaning that no significant similarities can be detected by BLAST).

### Derivation of group-specific amino acid similarity matrices

The reference sequence alignments from each SABmark group are used to derive a log-odds amino acid similarity matrix specific for this particular group (referred to as a family-specific matrix for an SUP group and as a fold-specific matrix for a TWI group). The matrices are derived using a modification of the approach suggested by Henikoff and Henikoff [20]. Given a set of reference pairwise sequence alignments from the SABmark group *k*, the elements of the group-specific amino acid similarity matrix *S_k _*are calculated as follows:

(1) $$s_{k}\left(i,j\right)=2\cdot \log_{2}\frac{q_{k}\left(i,j\right)}{e_{k}\left(i,j\right)}\cdot w_{k}+\left(1-w_{k}\right)\cdot v\left(i,j\right)$$

(2) $$q_{k}\left(i,j\right)=\frac{f_{k}\left(i,j\right)}{n_{k}}$$

(3) $$\begin{array}{c} e_{k}\left(i,i\right)=p_{k}\left(i\right)\cdot p_{k}\left(i\right)\\ e_{k}\left(i,j\right)=2\cdot p_{k}\left(i\right)\cdot p_{k}\left(j\right)\;\text{for}\;i\neq j \end{array}$$

(4) $$p_{k}\left(i\right)=q_{k}\left(i,i\right)+\frac{\underset{j\neq i}{\sum }q_{k}\left(i,j\right)}{2}$$

(5) $$w_{k}=1-10^{-\frac{n_{k}}{8000}}$$

Where *q_k_(i, j) *and *e_k_(i, j) *are the observed and expected frequencies of amino acid pair *(i, j) *in group *k*; *f_k_(i, j) *is the total number of amino acid pairs *(i, j) *in group *k*; *p_k_(j) *is the observed frequency of amino acid type *j *in group *k*; *n_k _*is the total number of amino acid pairs in group *k*; *v(i, j) *is the score for amino acid pair *(i, j) *from the VTML200 matrix [38]. The value of the constant, 8000, in Eq.5 was selected using a grid search as a value that results in the best overall performance of group-specific matrices.

In the final matrix, all elements *s_k_(i, j) *are rounded to the nearest integer. In order to account for sparse data in small SABmark groups, each element of the final matrix *S_k _*is calculated as the weighted combination of the group-specific score and the general score from the VTML200 matrix. As the total number of aligned amino acid pairs increases, the contribution of the group-specific score increases, whereas the contribution of the VTML200 score decreases. The VTML200 matrix is used in Eq.1 because it is the best performing general-purpose matrix (see 'Analysis of general-purpose matrices' for details).

### Matrix evaluation procedure

The main goal of this work is to compare the performance of various amino acid similarity matrices. This requires a quantitative framework for (1) evaluating the quality of pairwise sequence alignments obtained using a given similarity matrix, and (2) comparing how two different similarity matrices perform when used to align the same set of sequence pairs. The quality of a test alignment between sequences *i *and *j*, *Q(i, j)*, is evaluated by comparing this alignment to the reference SABmark structural alignment for the same pair of sequences, and is quantified by the average of two alignment accuracy measures, *f_D _*and *f_M _*[29]:

(6) $$Q\left(i,j\right)=\frac{f_{D}\left(i,j\right)+f_{M}\left(i,j\right)}{2}$$

(7) $$f_{D}\left(i,j\right)=\frac{n_{I}\left(i,j\right)}{l_{R}\left(i,j\right)}*100\%$$

(8) $$f_{M}\left(i,j\right)=\frac{n_{I}\left(i,j\right)}{l_{T}\left(i,j\right)}*100\%$$

Where *n_I_(i, j) *is the number of residue pairs aligned identically in the test and the reference alignments; *l_R_(i, j) *is the length of the reference alignment; *l_T_(i, j) *is the length of the test alignment. If the value of *Q(i, j) *equals 100%, it means that the test alignment and the reference alignment are identical. The test pairwise sequence alignments were obtained by using the Needlman-Wunsch algorithm [5] with the affine gap penalty function, G(m) = α + (m-1)*β, where *m *is the gap length, *α *is the gap initiation penalty, and *β *is the gap extension penalty. Gap penalties were optimized for each group/matrix as described below.

The performance of two given amino acid similarity matrices, *A *and *B*, in the pairwise alignment of sequences from a SABmark group *k *is analyzed by means of the two-sided paired t-test. In this approach, the pairs of sequences from group *k *are aligned using matrix *A *and the alignment quality score, *Q_A_*, is calculated for each pair using Eq.6. Then, the same sequence pairs from group *k *are aligned using matrix *B *and the alignment quality score, *Q_B_*, is calculated for each pair. After that, the distributions of alignment quality scores *Q_A _*and *Q_B _*are compared using the two-sided paired t-test. The outcome of the t-test is considered to be statistically significant if its *p*-value is less than 0.05. To avoid over-fitting, the alignment quality scores are obtained using a 3-fold cross-validation. In this procedure, each SABmark group is randomly partitioned into three non-overlapping sub-sets, with two sub-sets used for training and the remaining sub-set used for testing. The process is repeated three times, so that each sub-set is used for testing once. In the case of a general-purpose similarity matrix, "training" means optimizing gap penalties for this matrix. In the case of a similarity matrix specific for a SABmark group *k*, "training" means deriving the group-specific matrix itself and optimizing gap penalties for this matrix. "Testing" means using the matrix and optimized gap penalties obtained during the training step to align sequences from the test sub-set. During cross-validation, gap initiation, *α*, and gap extension, *β*, penalties for a given group *k *and a given similarity matrix *A *are optimized using the following grid search procedure: Sequences from the training set of group *k *are aligned using matrix *A*, and all possible combinations of integer gap penalties in range 1≤*α*≤50, 1≤*β*≤30 are tested; the combination (*α*, *β*) that results in the highest average quality score is selected as the best and is used with matrix *A *to align sequences from the test set of group *k*.

### Analysis of general-purpose matrices

In this section I compare the performance of existing general-purpose amino acid similarity matrices using the 3-fold cross-validation evaluation protocol described in the 'Matrix evaluation procedure' section. For this purpose, I chose two most popular series of matrices derived from multiple sequence alignments of evolutionary related sequences, the PAM matrices [21] and the BLOSUM matrices [20], along with matrices derived from structural superposition of remotely homologous proteins, BC-STR [24] and STROMA [39], a matrix derived by Gonnet et al [40], and the VTML200 matrix [38]. The PAM matrices were chosen because they are the oldest amino acid similarity matrices. The remaining matrices were chosen because, at some point in time, each of them was shown to be the top-performing matrix compared to others available at the same time period. The results of the pairwise comparison of the matrices on the SUP (Super-family) and TWI (Twilight) sub-sets are shown in Tables 1 and 2, respectively, and can be summarized as follows. In general, the VTML200 matrix tends to show a superior performance on the SUP groups. This is manifested by the largest number of SUP groups on which VTML200 performs significantly better when compared to other matrices. The second best-performing matrix is BLOSUM50, which ranks a little behind VTML200. The PAM family matrices, which are the oldest matrices used in this test, considerably underperform when compared to more recent matrices. This observation is consistent with the previously reported results [7,20]. No single general purpose matrix clearly outperforms other matrices on the TWI groups, the performance of VTML200 being similar to that of BLOSUM50/62 and BC-STR.

**Table 1:** The pair-wise comparison of general-purpose matrices on groups from the SUP sub-set.

	VTML	GONN	BL30	BL50	BL62	PAM120	PAM160	PAM250	BC-STR	STRM
**VTML**	0;0	55;5	98;12	28;15	23;11	81;4	83;4	78;8	44;16	59;5
**GONN**	5;55	0;0	41;16	11;47	13;42	27;10	27;10	33;8	16;43	24;19
**BL30**	12;98	16;41	0;0	9;77	12;82	18;35	21;28	20;29	4;64	12;47
**BL50**	15;28	47;11	77;9	0;0	12;10	57;6	53;3	59;6	28;15	56;11
**BL62**	11;23	42;13	82;12	10;12	0;0	60;7	51;9	56;11	23;21	43;8
**PAM120**	4;81	10;27	35;18	6;57	7;60	0;0	9;15	20;19	6;53	12;31
**PAM160**	4;83	10;27	28;21	3;53	9;51	15;9	0;0	13;16	10;49	10;29
**PAM250**	8;78	8;33	29;20	6;59	11;56	19;20	16;13	0;0	11;52	10;35
**BC-STR**	16;44	43;16	64;4	15;28	21;23	53;6	49;10	52;11	0;0	37;9
**STRM**	5;59	19;24	47;12	11;56	8;43	31;12	29;10	35;10	9;37	0;0

Matrices shown: VTML - VTML200 [38], GONN - Gonnet et al [40], BL30/50/62 - BLOSUM30/50/62 [20], PAM120/160/250 [21], BC-STR [24], STRM - STROMA [39]. A cell formed by the intersection of row i and column j shows the results of the pair-wise comparison of matrices (i, j), and contains two numbers: first is the number of groups on which matrix i performs significantly better than j, second is the number of groups on which matrix j performs significantly better than i.

**Table 2:** The pair-wise comparison of general-purpose matrices on groups from the TWI sub-set.

	VTML	GONN	BL30	BL50	BL62	PAM120	PAM160	PAM250	BC-STR	STRM
**VTML**	0;0	21;3	39;4	10;12	13;12	31;4	31;3	26;2	15;13	15;10
**GONN**	3;21	0;0	21;6	3;16	6;19	20;6	13;2	13;5	4;17	7;6
**BL30**	4;39	6;21	0;0	3;37	5;38	16;18	13;14	8;15	3;28	5;24
**BL50**	12;10	16;3	37;3	0;0	11;6	27;2	26;3	25;4	12;8	13;6
**BL62**	12;13	19;6	38;5	6;11	0;0	23;1	23;1	27;5	17;10	15;10
**PAM120**	4;31	6;20	18;16	2;27	1;23	0;0	11;10	12;12	2;25	6;18
**PAM160**	3;31	2;13	14;13	3;26	1;23	10;11	0;0	9;9	2;29	3;19
**PAM250**	2;26	5;13	15;8	4;25	5;27	12;12	9;9	0;0	4;24	5;17
**BC-STR**	13;15	17;4	28;3	8;12	10;17	25;2	29;2	24;4	0;0	11;5
**STRM**	10;15	6;7	24;5	6;13	10;15	18;6	19;3	17;5	5;11	0;0

Matrix names are the same as in Table 1. A cell formed by the intersection of row i and column j shows the results of the pair-wise comparison of matrices (i, j), and contains two numbers: first is the number of groups on which matrix i performs significantly better than j, second is the number of groups on which matrix j performs significantly better than i.

**Figure 1:**
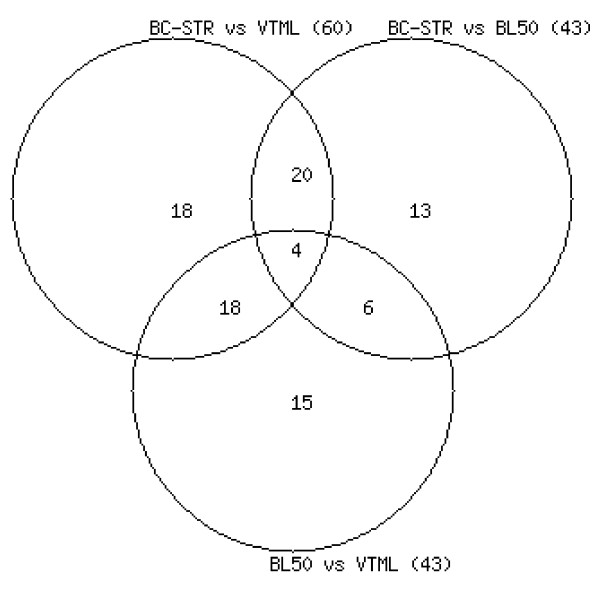
**The overlap between SUP groups that account for the significant differences**. The overlap between SUP groups that account for the significant differences observed in three pair-wise matrix comparisons: BC-STR vs. VTML200 (60 groups), BC-STR vs. BLOSUM50 (43 groups), and BLOSUM50 vs. VTML200 (43 groups).

The results reported in Tables 1 and 2 indicate that every tested matrix may perform significantly worse on some groups, depending on what other matrix it is compared to. This means that there is no single matrix that universally outperforms all other matrices on all SABmark groups. An important question is what are the identities of the groups that account for the observed differences, meaning if the same matrix is compared to different matrices, does it tend to perform significantly better/worse on the same set of groups or not? However, the results presented in Tables 1, 2 are for pairwise comparisons in which only two matrices are compared at a time and do not provide information about the identity of the groups that account for the observed differences. In order to answer this question, I conducted a detailed analysis of the overlap between SUP groups that account for significant differences observed in three pairwise comparisons: BC-STR vs. VTML200, BC-STR vs. BLOSUM50, and BLOSUM50 vs. VTML200. These three matrices were chosen because they were derived using different methodological approaches and source datasets, and at different time periods. The results of this analysis are summarized in the Vienn diagram shown in Figure 1. The main point of this figure is that the majority of groups do not overlap, which means that the same matrix may perform significantly better/worse on different groups, depending on what other matrix it is compared to. The most likely explanation of this observation is that the performance of each general-purpose matrix is significantly biased towards a certain set of protein families that were over- and under-represented in the dataset used to derive the matrix. When two matrices are compared, they perform similarly on families that were represented similarly in the two datasets used to derive these matrices, and differently on families that were over-represented in one dataset and under-represented in the other.

### Analysis of family- and fold-specific matrices

In this section test the performance of the group-specific approach. In this test, a group-specific similarity matrix is derived for each SABmark group according to Eq.1 and compared to the general-purpose matrices using the 3-fold cross-validation evaluation protocol described in the 'Matrix evaluation procedure' section. Only groups that have 10 or more reference alignments were used in this test because in smaller groups the total number of observed amino acid exchanges is not sufficient to derive a meaningful group-specific matrix. The results of this comparison on the SUP and TWI sub-sets are shown in Table 3 and can be summarized as follows. In the case of the SUP subset, the family-specific amino acid similarity matrices consistently tend to outperform all general-purpose matrices. The fraction of groups on which family-specific matrices perform significantly better ranges from 20.5% to 49.2%, depending on which general purpose matrix they are compared to. For instance, when the family-specific approach is compared to the two top-performing general-purpose matrices, VTML200 and BLOSUM50, it yields significantly better alignments for 50 (20.5%) and 74 (30.3%) groups, respectively, whereas VTML200 and BLOSUM50 produce significantly better alignments for only 5 (2%) and 6 (2.5%) groups, respectively. It is reasonable to expect that when more reference alignments become available for the under-represented SABmark families, the performance of the family-specific approach can be improved further. In the case of the TWI subset, the fold-specific approach performs only slightly better than the VTML200 matrix. This is not surprising because each TWI group consists of proteins that share the same fold, but do not share clear evolutionary relationships. It has been shown previously that pairwise sequence alignment tends to perform poorly on such proteins from the so-called "twilight zone" [37,41]. Thus, using protein family-specific similarity matrices can significantly improve the quality of alignment of homologous sequences with low to moderate degrees of sequence identity, but using fold-specific matrices can only marginally improve sequence alignment of structurally-similar proteins from the "twilight zone".

**Table 3:** The pair-wise comparison of group-specific matrices vs. general-purpose matrices.

	VTML	GONN	BL30	BL50	BL62	PAM120	PAM160	PAM250	BC-STR	STRM
**FSM-SUP**	50;5	86;1	120;5	74;6	63;0	106;3	110;4	114;3	67;7	93;0

**FSM-TWI**	15;2	20;1	41;3	21;9	19;4	36;0	37;1	35;0	21;4	24;1

**Adaptive** **FSM-SUP**	35;6	70;2	103;7	50;9	45;3	90;4	96;4	98;4	60;14	73;1

Matrix names are the same as in Table 1. Abbreviations: FSM-SUP - family-specific matrices tested on groups from the SUP sub-set. FSM-TWI - fold-specific matrices tested on groups from the TWI sub-set. Adaptive FSM-SUP - the adaptive family-specific matrix selection procedure tested on the groups from the SUP sub-set. The results shown are for groups with 10 or more reference alignments (244 groups in the SUP sub-set, 131 groups in the TWI sub-set). A cell formed by the intersection of row i and column j shows the results of the pair-wise comparison of matrices (i, j), and contains two numbers: first is the number of groups on which matrix i performs significantly better than j, second is the number of groups on which matrix j performs significantly better than i.

**Figure 2:**
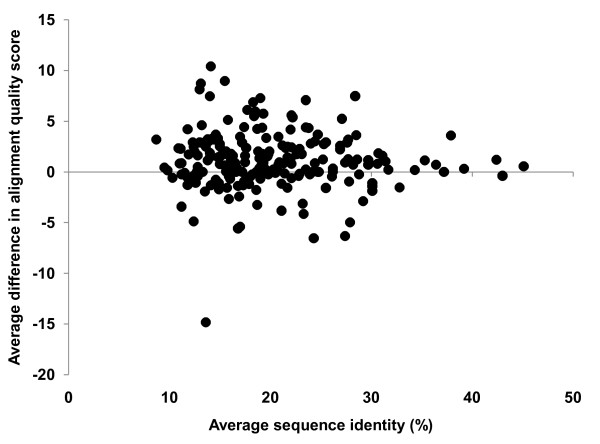
**The scatter plot of the average difference in the alignment quality score between the family-specific matrices and the VTML200 matrix**. Each dot represents one group from the SUP subset. A positive value of the average difference indicates that the family-specific similarity matrix performs better than VTML200. The Pearson's correlation coefficient calculated using the data points from this plot is 0 (no correlation).

**Figure 3:**
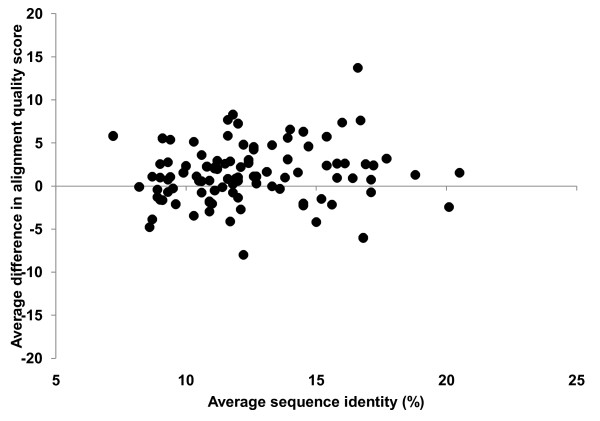
**The scatter plot of the average difference in the alignment quality score between the fold-specific matrices and the VTML200 matrix**. Each dot represents one group from the TWI subset. A positive value of the average difference indicates that the fold-specific similarity matrix performs better than VTML200. The Pearson's correlation coefficient calculated using the data points from this plot is 0 (no correlation).

An important question is how similarities between aligned sequences affect the performance of group-specific matrices. Do these matrices tend to perform better than general-purpose matrices on groups that contain many similar or many dissimilar sequences? In order to answer this question, I calculated two parameters for each SABmark group: (1) the average difference in the quality score (Eq.6) between alignments obtained with the group-specific matrix and with the VTML200 matrix, and (2) the average sequence identity. The scatter plots of these parameters for the SUP and TWI sub-sets are shown in Figures 2 and 3. The main point of these plots is that there is no correlation between the average difference in alignment quality score and the average sequence identity in both SUP and TWI sub-sets. This means that the group-specific approach tends to perform better on proteins with both low and moderate sequence identity.

Finally, I studied how the family-specific matrices differ from the original VTML200 matrix, and how the family-specific matrices that perform better than VTML200 differ from the family-specific matrices that do not. The fold-specific matrices were not studied in detail because they do not appear to improve alignment quality significantly. First, the family-specific matrices were compared to the original VTML200 matrix by using the average difference between matrix elements, *D*1*(i, j)*:

(9) $$D1\left(i,j\right)=\frac{\overset{N}{\underset{k=1}{\sum }}\left[s_{k}\left(i,j\right)-v\left(i,j\right)\right]}{N}$$

**Figure 4:**
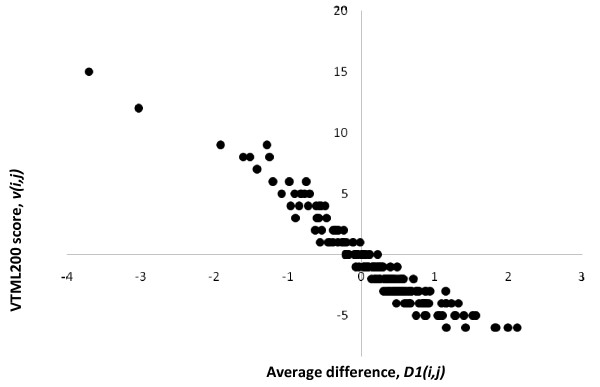
**The scatter plot of the average difference between the elements of the family-specific matrices and the VTML200 matrix**. Each dot represents one amino acid pair (210 total). A positive value of the average difference *D*1*(i, j) *(Eq.9) indicates that amino acid pair (*i, j*) tends to receive a higher score in the family-specific matrices compared to VTML200 and *vice versa*.

Where *s_k_(i, j) *is the score for amino acid pair (*i, j*) from the family-specific matrix *k*; *v(i, j) *is the score for amino acid pair (*i, j*) from the VTML200 matrix; *N *is the total number of family-specific matrices. The scatter plot of the average distance *D*1*(i, j) vs*. the original VTML score *v(i, j) *for all 210 amino acid pairs is shown in Figure 4. This plot indicates that identical (WW, CC, PP, YY, *etc*.) or similar (KR, YF, VI, DN, *etc*.) amino acid pairs that receive high scores in VTML200 tend to receive lower, but still positive, scores in the family-specific matrices. On the other hand, dissimilar amino acid pairs (WE, FD, IG, YP, *etc*.) that receive low scores in VTML200 tend to receive higher, but still negative, scores in the family-specific matrices. The higher the magnitude of the original VTML200 score, the more it tends to be affected in the family-specific matrices.

Second, the family-specific matrices that perform significantly better than VTML200 were compared to the family-specific matrices that perform similarly to VTML200 by using the difference between the average matrix elements, *D*2(*i, j*):

(10) D2(i,j)=BP(i,j)-SP(i,j)

(11) $$BP\left(i,\;j\right)=\frac{\overset{NB}{\underset{k=1}{\sum }}SB_{k}\left(i,j\right)}{NB}$$

(12) $$SP\left(i,\;j\right)=\frac{\overset{NS}{\underset{k=1}{\sum }}SS_{k}\left(i,j\right)}{NS}$$

Where *SB_k_(i, j) *is the score for amino acid pair (*i, j*) from the *k*^th ^family-specific matrix that performs better than VTML200; *SS_k_(i, j) *is the score from the *k*^th ^family-specific matrix that performs similarly to VTML200; *NB *is the number of matrices that perform better than VTML200; *NS *is the number of matrices that perform similarly to VTML200. The family-specific matrices that perform significantly worse than VTML200 were not used because there were only 5 of them. To remove the cases that show a trend toward statistical significance, matrices that perform similarly to VTML200 were defined as the ones that received the *p*-value greater than 0.1 during the matrix evaluation procedure (156 matrices total).

**Figure 5:**
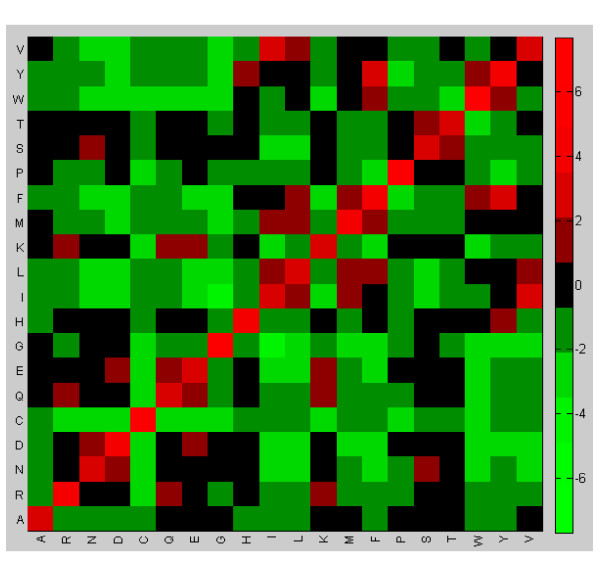
**The heat map of the elements of the matrix *BP *(Eq.11)**. The cell (*i, j*) of the heat map represents the element *BP*(*i, j*) (Eq.11). Red colors denote the positive values of *BP*(*i, j*). Green colors denote the negative values of *BP*(*i, j*).

**Figure 6:**
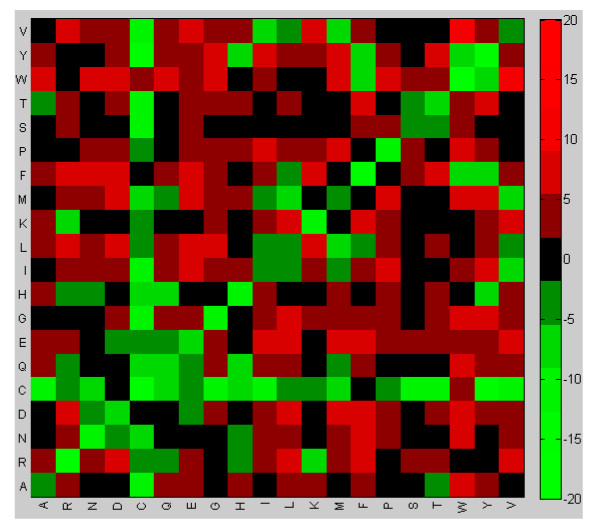
**The heat map of the elements of the matrix *D*2 (Eq.10)**. The cell (*i, j*) of the heat map represents the element *D*2(*i, j*) (Eq.10) multiplied by 10. Red colors denote the positive values of *D*2(*i, j*). Green colors denote the negative values of *D*2(*i, j*).

The heat maps summarizing the values of the elements of matrices *BP *and *D*2 are shown in Figures 5, 6. These heat maps indicate that the family-specific matrices that perform significantly better than VTML200 tend to follow the general rules and assign positive scores to identical/similar pairs and negative scores to dissimilar pairs (Figure 5). However, the difference between the family-specific matrices that perform significantly better than VTML200 and those matrices that do not is that the former tend to assign lower scores to all identical pairs and to many similar pairs (YF, IV, TS, KR, DN, ED, *etc*.), while assigning higher scores to many dissimilar pairs (such as hydrophobic-hydrophilic pairs) (Figure 6). Notably, most cysteine-containing pairs, including CC, receive lower scores in the family-specific matrices that perform significantly better than VTML200, except for pairs CW, CF, and CD. To summarize, the family-specific approach, on average, tends to make the alignment of identical and similar pairs less favorable and the alignment of dissimilar pairs more favorable. These tendencies are particularly profound in the family-specific matrices that perform significantly better than VTML200. In general, these observations conform to the empirical expectation that a good quality alignment of divergent members of the same protein family must correctly align only a limited number of conserved core residues specific for this family and accommodate a large number of amino acid substitutions. This implies decreasing the scores for certain identical/similar pairs and increasing the scores for certain dissimilar pairs in the family-specific matrix.

### Adaptive protein family-specific alignment

The analysis of the performance of the group-specific matrices described above is based on the knowledge of the group membership for each sequence used for testing, meaning that a correct group-specific matrix is always selected for each alignment by default. However, in the real world we do not always know in advance what particular family a given pair of homologous sequences belongs to. How to select an appropriate family-specific matrix in this case? I used BLAST [2] sequence similarity search to design an adaptive alignment procedure that automatically selects an appropriate family-specific similarity matrix for aligning a pair of input sequences. In this approach, a given input sequence is used as a query and the entire SUP subset (except for the sequences used for testing) is used as a sequence database to run a BLASTP search. If this search returns one or more hits with E-value less than 0.05, then the family membership of the database sequence with the smallest E-value is assigned to the input sequence. For a pair of input sequences two independent BLASTP searches are run, one for each sequence, and a similarity matrix for aligning this pair is selected using the following rules. If both sequences are assigned to the same family *k*, then the family-specific matrix *k *and the optimized gap penalties for this matrix are used to align these sequences. If the sequences are assigned to different families or if at least one sequence is not assigned to any family because of the absence of significant hits, then VTML200 (the best performing general-purpose matrix) with the gap initiation penalty of -15 and the gap extension penalty of -1 is used to align these sequences. These default gap penalties were determined by the grid search procedure as the best-performing general-purpose combination for VTML200 - among all combinations tested, (-15,-1) results in the highest average alignment quality score calculated for the dataset that contains all SUP groups pooled together.

The results of the assessment of the performance of the adaptive alignment procedure on the SUP sub-set, as determined by the 3-fold cross-validation, are shown in Table 3. The procedure was not tested on the TWI sub-set because sequences in this sub-set do not share similarities that can be detected by BLASTP. Overall, the adaptive procedure provides a correct family assignment for 53.68%, no assignment for 46.31%, and incorrect assignment for 0.01% of all sequence pairs. One potential direction for further improving the matrix selection procedure is to use a sequence comparison program which is more sensitive and specific than BLASTP. Nevertheless, even despite the observed incomplete family assignments, the adaptive alignment procedure significantly outperforms general-purpose matrices. For instance, the adaptive procedure significantly outperforms VTML200 on 35 (14.3%) families and BLOSUM50 on 50 (20.5%) families. Thus, it can be used for a completely automated alignment of homologous protein sequences using the proposed family-specific similarity matrices in the absence of a prior knowledge of what specific protein family these sequences belong to.

## Conclusions

1. I utilized a large set of reference alignments obtained from the structural superposition of homologous and analogous proteins to design a quantitative statistical framework for the comparative evaluation of the performance of alignment scoring functions in the pairwise global sequence alignment. This framework was applied to study how the existing general-purpose amino acid similarity matrices perform on individual protein families and structural folds and to compare them to the family-specific and fold-specific similarity matrices derived in this work.

2. Among all the general-purpose matrices tested, VTML200 is the best-performing one. It produces global pair-wise sequence alignments most similar to the reference alignments derived from structural superposition. However, the results of this work suggest that the performance of each general-purpose matrix may be significantly biased towards a certain set of protein families. Even the top-performing general-purpose matrix cannot universally outperform other matrices on all protein families and all folds.

3. Using protein family-specific similarity matrices and optimized gap penalties can significantly improve the quality of alignment of homologous sequences compared to the traditional sequence alignment based on a single general-purpose similarity matrix. However, using fold-specific matrices can only marginally improve sequence alignment of proteins that share the same structural fold but do not share a well-defined common evolutionary origin.

4. I presented an adaptive alignment procedure that automatically selects an appropriate amino acid similarity matrix and optimized gap penalties based on the properties of the sequences being aligned. This procedure does not require a manual assignment of evolutionary relationships and can be used for an automated optimized alignment of homologous protein sequences in the absence of a prior knowledge of what protein family these sequences belong to. The current limitation of the presented adaptive alignment procedure is that it is directly applicable only to single-domain proteins or to individual domains from multi-domain proteins. This limitation will be addressed in the future.

## Availability and requirements

The protein family-specific amino acid similarity matrices derived in this work and the associated optimized gap penalties, along with the optimized family-specific gap penalties for the VTML200 and BLOSUM50 matrices, are available on-line at http://taurus.crc.albany.edu/fsm.

## Competing interests

The author declares that they have no competing interests.

## Authors' contributions

IBK conceived of the study, performed it, and drafted the manuscript. All authors read and approved the final manuscript.
